# The clinical relevance of grip strength: A narrative exploration

**DOI:** 10.6026/9732063002001291

**Published:** 2024-10-31

**Authors:** Swati Tapaswi Kanna, Shivangi Saxena, Reema Reji, Rishabh Jain, Ruchi Kothari, Mayur Wanjari, Ravi Sangoi, Krisha Jain

**Affiliations:** 1 Department of Medicine, Royal Albert Edward Infirmary, Wigan, United Kingdom; 2 Department of Medicine, Rajarajeshwari Medical College and Hospital, India; 3 Department of Paediatrics, KM Cherian Institute of Medical Sciences, Kallissery, Chengannur, India; 4 Department of Internal Medicine, JSS Medical College, Mysore, India; 5 Department of Physiology, Mahatma Gandhi Institute of Medical Sciences, Sevagram, Wardha, Maharashtra, India; 6 Department of Research, Datta Meghe Institute of Higher Education & Research (DMIHER), Sawangi, Maharashtra, India; 7 Department of Internal Medicine, Government Medical College, Baramati, India

**Keywords:** Hand Grip Strength (HGS), isometric strength, diabetes, rehabilitation, upper limb, metabolic syndrome

## Abstract

Hand grip strength (HGS) is a well-established measure of musculoskeletal function and integrity of the upper limb. Traditionally used in rehabilitation, its utility extends too many clinical conditions in primary care practice. Therefore, it is of interest to explore the diverse applications of HGS in medical practice. In osteoporosis, HGS predicts bone density and risk of fracture. In osteoarthritis, it serves as a benchmark for functional impairment. As a nutritional marker, HGS reflects malnutrition and predicts postoperative outcomes. In metabolic syndrome and chronic diseases, HGS relates to conditions like diabetes, hypertension and cardiovascular risk. Further, additional values of HGS are in the oncology field in evaluating nutritional status and survival in patients with cancer. Its relevance in kidney diseases and conditions such as carpal tunnel syndrome further supports its broad clinical utility. Particularly, it has highlighted the potential which HGS may possess as simple yet effective means for evaluating health and guiding interventions across diverse clinical scenarios.

## Background:

Hand grip strength (HGS), a crucial component of human function, is easily assessed by measuring the amount of static force the hand can apply to compress a transducer [1]. It is a dependable and established objective parameter to evaluate the functional integrity of the upper extremity as a component of the musculoskeletal system.

## Clinical relevance of HGS evaluation:

Until now, the evaluation of HGS is of great importance in the assessment of upper limb impairment, to measure the baseline deficiency in hand muscle power, set treatment goals, to monitor progress during rehabilitation, to document the effectiveness of various treatment strategies and to assess patient's ability to return to employment. But over the years, it has been documented in the extensive literature that the extent of implications of HGS is far reaching and encompassing a plethora of clinical conditions where its assessment can work wonders! To have a closer look at its broad spectrum of applications, the review now highlights the significant findings of prominent research works performed in this arena.

## HGS and Osteoporosis:

A cross-sectional study by Lin *et al.* (2020) aimed to investigate the suitability of HGS in predicting the risk of osteoporosis in Asian adults. All 1007 participants were evaluated for HGS as measured by a digital dynamometer, which was significantly related to bone density and bone microarchitecture. Specifically, for women, a HGS of 21.9 kg was associated with sensitivity of 59% and specificity of 59% in predicting osteoporosis. In men, a threshold of 28.7 kg had sensitivity of 66% and specificity of 78% [2]. A cross-sectional study by Nagai *et al.* (2021), enrolled 349 osteoporotic females, all aged >65 years and revealed in age-matched multivariate analysis, significant, independent associations between high-risk of fall injury with both vitamin D deficiency and low HGS [3].

## HGS and Osteoarthritis (OA):

A prospective case-control study by Villafañe *et al.* (2017), with 57 OA cases and 53 controls used a two-way ANOVA test to establish quantifiable benchmarks through minimal clinically important difference (MCID) scores for grip, tip and tripod pinch strength [4].

## Grip strength as a nutritional marker:

A total of 86 malnourished patients undergoing major abdominal surgery, were administered oral nutritional supplements during their hospital stay by Keele *et al.* (1997). Using HGS as the outcome indicator, control patients exhibited a significant decline in HGS during their hospital stay, while intervention patients experienced a temporary drop in HGS at day 3, which returned to preoperative levels by discharge, indicating the potential protective effect of nutritional intervention [5]. Beattie *et al.* (2000) studied the effects of a 10-week oral nutritional supplement intervention on 101 malnourished surgical patients. The findings revealed that postoperative reductions in HGS were less pronounced in the intervention group, with significantly better HGS values at the end of the study period compared to controls [6]. A study by Mahalakshmi *et al.* (2004), established Maximal Grip Strength (MGS) as a simple, effective bedside test that can complement clinical scoring to predict postoperative complications, proving to be a better indicator than serum albumin [7, 8]. Edington *et al.* (2004) found significant improvements in HGS in the intervention group during an eight-week supplementation period, with notable differences from the control group [8]. Price *et al.* (2005) reported a greater increase in HGS among the intervention group (13.9%) compared to the control group (7.2%) [9]. A review by Norman *et al.* (2011) explored the potential of HGS as a marker of nutritional status in Germany. They concluded that muscle function, as assessed by HGS, reacts early to nutritional deprivation thereby reiterating impaired grip strength is an indicator of increased postoperative complications, increased length of hospitalization, higher rehospitalisation rate and decreased physical status [10].

Another study by Lombardo *et al.* (2021), found higher prevalence of chronic diseases (diabetes and hypertension) In women with lower BMI-adjusted HGS suggesting it to be a useful measure of muscle strength and its independent association with diabetes in this particular age group [11]. Using the Cox proportional hazards model, the prediction of outcomes in patients with ischemic diabetic foot ulcers (IDFU), a study by López-Valverde *et al.* (2023), reported decreased HGS mean as a significant parameter for predicting mortality [12]. Recently Cifuentes-Amigo *et al.* (2024) in 847 participants indicated that HGS had a higher correlation with nutritional status than knee extensor strength, particularly right HGS (r: -0.40) [13].

## HGS in metabolic syndrome:

Mainous *et al.* (2015) examined the relationship between HGS and diabetes and hypertension in healthy-BMI adults aged 20 years or more. Individuals with undiagnosed diabetes had significantly lower HGS compared to those without diabetes. Similarly, individuals with diagnosed diabetes also had lower HGS than those without diabetes. Mean HGS was lower among individuals with undiagnosed hypertension compared to those without hypertension. Individuals with diagnosed hypertension also had lower HGS than those without hypertension. HGS was thus lower in adults with metabolic co-morbidities [14]. A study by Wong *et al.* (2022), explored a novel composite measure MSI or the Muscle Strength Index to find associations with diabetes. This MSI score was derived from HGS and the timed 5-repitition chair stand test (RCS) both. It was more strongly associated with diabetes then poor HGS and just RCS alone, individually [15]. In a recent study by Hamasaki & Yanai (2023), HGS was inversely and independently associated (r = - 0.270, p = 0.006) with augmentation index, a measure of systemic arterial stiffness. Thereby concluded a way of intervening early to prevent cardiovascular mortality in HGS diminished type 2 diabetes patients [16].

## HGS in nutritional oncology:

In a study by Kilgour *et al.* (2013), involving 203 patients with non-small cell lung and gastrointestinal cancers, participants were categorized into three HGS percentiles (≥50th, 25th, ≤10th). A multivariate regression analysis revealed that as compared to patients in the ≥50th percentile, those in the ≤10th percentile had lower BMI, and shorter survival [Hazard Ratio: 3.2 (95% CI, 2.0-5.1)]. Specifically, patients in the lowest HGS percentile had a BMI that was 2.5 kg/m^2^ lower, a threefold increase in mortality risk, suggesting HGS is independently associated with survival in cancer patients [17]. A cross-sectional study by Alkan *et al.* (2018), attempted to assess malnutrition among 104 cancer [52 gastrointestinal system (GIS) and 52 non-GIS cases] patients, thereby evaluate their nutritional status. While significant association was seen between HGS and LBM (p= 0.000), an insignificant negative association was seen between HGS and the Patient-Generated Subjective Global Assessment (PG-SGA score) (p= 0.071) [18] whereas Valente *et al.* (2019) showed significant correlations with conventional anthropometric variables and the PG-SGA score, indicating their utility as reliable, complementary methods for assessing nutritional risk in cancer patients [19]. A study by Laura *et al.* (2022) identified HGS-evaluated nutritional status as a complement to PS-SGA in both, improving and predicting clinical outcomes of cervical cancer patients undergoing GI toxic chemotherapy [20].

## Vitamin D and HGS:

A case control study by Dhanwal *et al.* (2013) explored the relationship between Vitamin D deficiency and HGS. 95 hip fracture patients and 95 matched controls were selected. HGS was estimated using a hand dynamometer. HGS was also lower in this group in comparison with the control group. They could also find a positive correlation between vitamin D levels and HGS [21]. A study by Fu *et al.* (2022), observed in 108 haemodialysis dependent participants that Vitamin D levels have a significant positive association with HGS [22].

## HGS in kidney diseases:

Amparo *et al.* (2013), through univariate analysis, found that malnutrition-inflammation score (MIS) and HGS are negatively correlated even in non-dialysis-dependent chronic kidney disease individuals [23]. A prospective cohort study by Matos *et al.* (2014) employed Cox regression models to conclude that the hazard of death was significantly higher in both, males and females with lower HGS. This study reiterated how HGS can possibly predict all-cause mortality in haemodialysis patients [24]. An observational cohort study by Birajdar *et al.* (2019) on 83 patients on maintenance haemodialysis revealed the association of HGS with malnutrition in such patients. Majority of the male subjects had a weaker HGS than that of age-matched control. A significant association of serum creatinine and HGS was found [25].

## Carpal tunnel syndrome and HGS:

A study by Singh & Srivastava (2020) aimed at finding association of HGS with carpal tunnel syndrome (CTS) in occupational workers. It included 60 workers (15 each from four different occupations). Grip strength was measured by handheld dynamometer. Individuals with CTS experienced a noticeable decline in HGS, proportional to the severity of CTS [26]. Hand grip strength (HGS) is a key indicator of muscle function, overall physical capability, and health, particularly in assessing sarcopenia in the ageing population [27].

## Conclusion:

The latest advancements in the application of HGS in medical and research settings are reviewed. It is clearly evident by casting a cursory glance over the expansive work conducted on the broad ranging spectrum of clinical conditions wherein HGS can be employed that it would not be an overstatement to make that the assessment of HGS is extensively utilized in both clinical practice and research.
